# *In Vivo* Detection of Perinatal Brain Metabolite Changes in a Rabbit Model of Intrauterine Growth Restriction (IUGR)

**DOI:** 10.1371/journal.pone.0131310

**Published:** 2015-07-24

**Authors:** Rui V. Simões, Emma Muñoz-Moreno, Rodrigo J. Carbajo, Anna González-Tendero, Miriam Illa, Magdalena Sanz-Cortés, Antonio Pineda-Lucena, Eduard Gratacós

**Affiliations:** 1 BCNatal—Barcelona Center for Maternal-Fetal and Neonatal Medicine (Hospital Clínic and Hospital Sant Joan de Deu), Fetal i+D Fetal Medicine Research Center, IDIBAPS, University of Barcelona, Centre for Biomedical Research on Rare Diseases (CIBER-ER), Barcelona, Spain; 2 Structural Biochemistry Laboratory, Centro de Investigación Príncipe Felipe, Valencia, Spain; INRA, FRANCE

## Abstract

**Background:**

Intrauterine growth restriction (IUGR) is a risk factor for abnormal neurodevelopment. We studied a rabbit model of IUGR by magnetic resonance imaging (MRI) and spectroscopy (MRS), to assess *in vivo* brain structural and metabolic consequences, and identify potential metabolic biomarkers for clinical translation.

**Methods:**

IUGR was induced in 3 pregnant rabbits at gestational day 25, by 40–50% uteroplacental vessel ligation in one horn; the contralateral horn was used as control. Fetuses were delivered at day 30 and weighted. A total of 6 controls and 5 IUGR pups underwent T2-w MRI and localized proton MRS within the first 8 hours of life, at 7T. Changes in brain tissue volumes and respective contributions to each MRS voxel were estimated by semi-automated registration of MRI images with a digital atlas of the rabbit brain. MRS data were used for: (i) absolute metabolite quantifications, using linear fitting; (ii) local temperature estimations, based on the water chemical shift; and (iii) classification, using spectral pattern analysis.

**Results:**

Lower birth weight was associated with (i) smaller brain sizes, (ii) slightly lower brain temperatures, and (iii) differential metabolite profile changes in specific regions of the brain parenchyma. Specifically, we found estimated lower levels of aspartate and N-acetylaspartate (NAA) in the cerebral cortex and hippocampus (suggesting neuronal impairment), and higher glycine levels in the striatum (possible marker of brain injury). Our results also suggest that the metabolic changes in cortical regions are more prevalent than those detected in hippocampus and striatum.

**Conclusions:**

IUGR was associated with brain metabolic changes *in vivo*, which correlate well with the neurostructural changes and neurodevelopment problems described in IUGR. Metabolic parameters could constitute non invasive biomarkers for the diagnosis and abnormal neurodevelopment of perinatal origin.

## Introduction

Intrauterine growth restriction (IUGR) occurs in 5–10% of gestations and, according to current evidence, is mainly caused by placental insufficiency. Compromised blood supply can ultimately lead to sustained hypoxemia and undernutrition of the growing fetus [1]. Consequently, IUGR is associated with an increased risk of adverse perinatal outcome [2] and suboptimal neurodevelopment [3]. Clinical studies have shown pre- [4,5] and post-natal [6,7] structural changes in IUGR brains, together with neurodevelopmental impairment during neonatal life [4,5] and early infancy [6,7]. Perinatal diagnosis and clinical interventions, aimed at improving neurobehavioral outcome in IUGR [8], are hampered by a limited understanding of the pathophysiological basis and a lack of biomarkers of brain reorganization occurring under IUGR.

Since gross morphologic changes in tissues have a metabolic origin, recent clinical studies have explored *in vivo* molecular biomarkers of IUGR based on changes in fetal brain metabolic profiles, using non-invasive proton magnetic resonance spectroscopy (MRS) [9,10]. However, the information obtained so far is hampered by the inherent sensitivity limitations of clinical magnetic fields and the unrestrained movements of the fetal head. An approach to improve the identification of metabolic biomarkers of IUGR is the use of pre-clinical animal models, allowing the application of stronger magnetic fields and specific holders for animal restraining, thus providing an opportunity to increase the sensitivity of the measurements.

A rabbit model of IUGR, based on the partial ligation of placental vessels [11,12], has been shown to closely reproduce cardiovascular clinical features of this condition during fetal life [13], as well as structural changes in the brain and neurobehavioral impairments at the neonatal period [12,14] and at pre-adolescent equivalent age [15,16]. Recently, mass spectrometry (LC-MS) analysis of *post-mortem* brain samples obtained from this model at the time of birth revealed specific metabolome changes associated with IUGR, suggesting alterations in several cellular parameters such as neuronal viability, energy metabolism, and oxidative stress [17]. However, the use of brain tissue obtained after the animal sacrifice might introduce a bias in the interpretation of which metabolites can be readily detected *in vivo*, since potential *post-mortem* ischemia effects cannot be ruled out.

Therefore, this study is aimed at using the same rabbit model of IUGR to investigate *in vivo* the metabolite profile changes in different brain regions at the time of birth.

## Methods

### Animal model of IUGR

All the experiments and animal handling procedures in this study were approved by the *Animal Experimental Ethics Committee* of the University of Barcelona (permit number: 206/ 10–5440) and performed according to the local guidelines. At gestation day 25, IUGR was induced in three New Zealand rabbits (Granja San Bernardo, Navarra, Spain), as reported previously [12]. Prior to surgery, progesterone (0.9 mg/kg i.m.) and penicillin (G, 300,000 IU i.v.) were administered to each pregnant rabbit for tocolysis and prophylaxis, respectively. Anesthesia was induced by intramuscular injection (ketamine-xylazine: 35 mg/kg—5 mg/kg) and mantained by endovenous perfusion (ketamina-xylazine: 2 mg/kg—0.6 mg/kg). A local analgesic was also administered along the abdominal midline (bupivacaine: 0.5%, 5 mL s.c.), followed by laparotomy. Both uterine horns were exposed and the gestational sacs counted. Each horn was randomly assigned as IUGR or control, assuring at least four live fetuses in the former to account for the expected mortality rates associated with this model of IUGR (36.2% [15]). The control horn was returned to the abdominal cavity. In the IUGR horn, the uteroplacental vessels of all gestational sacs were partially ligated (40–50%, visual inspection) using silk sutures 4/0, while continuously rinsing with warm Ringer lactate solution. After the procedure, the abdomen was sutured in two layers (silk 3/0) and animals were kept under a warming blanket until becoming active. Analgesia was administered subcutaneously at that point (buprenorphine: 0.05 mg/kg) and animals were returned to their cages. Analgesia was also added to the drinking water over the following 48h (buprenorphine: 0.05 mg/mL) and pregnant rabbits were controlled daily until gestational day 30 (5 days after surgery). At that point, all fetuses were delivered by cesarean section, performed under the same anesthetic procedure. Living and stillborn fetuses were counted, and living newborns and their placentas were weighed. The relative position of each fetus was determined as the ratio of its position in the respective horn (starting from the ovaric end) to the total number of fetuses in the same horn. The mothers were sacrificed during anesthesia, with pentobarbital (200mg/kg). Due to time restrains, two controls and two IUGR newborns from each litter were selected for magnetic resonance (MR) examination. In each litter, animals were selected based on their birth weight, as the most representative of the average weight in the respective horn. The pups were wrapped in the mother's fur and transported in warm ventilated box to the 7T MR room (15–20 min away from the animal facility). The remaining animals were sacrificed at birth, by decapitation.

### Metabolite basis sets for in vivo MRS

The metabolite basis sets used for quantification of *in vivo* MRS data were generated based on additional *in vitro* and *ex vivo* MRS experiments with control animals from different mothers, as detailed in **S1 Methods** section. Briefly, these experiments consisted in: A) High-Resolution Nuclear Magnetic Resonance (NMR) analysis of *in vitro* metabolite extracts from *post-mortem* frozen brain sections (3 control brains); B) High Resolution Magic Angle Spinning (HR-MAS) of *ex vivo* brain tissue biopsies obtained after focused microwave fixation (3 additional control brains) (**S1 Fig**). The total creatine levels determined from HR-MAS experiments were used as reference for the quantification of *in vivo* MRS data.

### *In vivo* acquisition of MRI/MRS

MR was carried out on a 7T horizontal magnet (Bruker BioSpec, Ettlingen, Germany) equipped with a four channel surface phased array RF coil for small animal's brain and running with *ParaVision* 5.1. Animals were kept anesthetized with 1–2.5% isoflurane in a 30/70% O_2_/N_2_O mixture, maintaining their respiratory frequency between 30–60 breaths/min. The body temperatures were controlled with a rectal probe and a recirculating heated water blanket. After the scout localizers, T2-w spin-echo RARE (*Rapid Acquisition with Relaxation Enhancement* [18]) images were obtained in the three orthogonal planes with the following parameters: 4500ms repetition time (TR), 11ms echo time (TE), 22x22mm field of view, 1mm in plane resolution, 1mm slice thickness, 8 turbo factor, and 2min24sec acquisition time. These images were used for sequentially positioning a PRESS (*Point Resolved Spectroscopy* [19]) voxel in three different brain tissues: cortex, 1.3x2.0x4.0mm (10.4 μL); hippocampus, 1.6x4.3x1.5mm (10.3 μL); and striatum, 2.1x1.4x3.6mm (10.6 μL). ^1^H-MRS data were acquired from each of these voxels with the following parameters: 2500msTR, 12ms TE, VAPOR (*Variable Pulse Power and Optimized Relaxation Delays* [20]) partial water-suppression (200Hz bandwidth), outer volume suppression (3 mm slice thickness), 256 averages, 4 dummy scans, and 10min50sec acquisition time. A reference water scan (without VAPOR) was also acquired prior to the water-suppressed, with the same parameters but only 8 averages. After the MR studies, animals under isoflurane anesthesia were immediately sacrificed by decapitation.

### Processing of *in vivo* MRI data

Parcellation of the different brain tissues in T2-w images was performed to estimate their respective volumes and relative contributions to each MRS voxel. For this, we used an adapted version of our digital rabbit brain atlas, developed according to T1-w images of e*x vivo* rabbit brains at postnatal day 70 [21]. Thus, the original T1 atlas template was elastically registered to one of our *in vivo* T2-w images, at postnatal day 0 by a multimodal version of the consistent block matching algorithm [22,23], implemented in-house using C++ and ITK libraries (www.itk.org). Then, the elastic transformation was applied to the region labeled image, obtaining an initial version of the parcellation that was reviewed and manually corrected by an expert, as before [21]. Some of the tissues present in the adult brain were still not identifiable on our T2-w images of the neonatal brain and were removed (such as claustrum, fornix, forebrain), and an additional label for cerebrospinal fluid was included. The final region map was used as reference atlas for brain parcellation. The T2-w image was used as template and elastically registered to the remaining subjects using the block matching algorithm [22,23], and automatic parcellations obtained by applying the transformation to the region map. All the parcellations were carefully reviewed and manually corrected if necessary, using ITK-SNAP v3.2 [24]. Finally, each MRS voxel used was spatially overlapped with the parceled T2-w image to compute its composition, i.e. respective brain tissue volumes and relative contributions, using *MATLAB R2010a* scripts developed in-house.

### Processing of *in vivo* MRS data

#### Local brain temperatures

The partially suppressed water peak was used for estimating the local brain temperatures in each voxel, using *jMRUI* v4.0 [25]. Brain temperatures were calculated based on the chemical shift displacement of the residual water peak with temperature [26] and using the total choline peak (3.21 ppm) as reference, as reported before in the mouse brain [27]:

Temperature(°C)=−82.33×(Dist+1.21)+255.94 (1)

being Dist the distance (ppm) between the residual water peak and the choline peak.

#### Metabolite Quantifications

MRS-detectable metabolites were quantified with *LCModel* v6.3 [28], using a basis set that included simulations of the metabolites identified in brain tissue samples of the same animal model by high resolution NMR and *ex vivo* HR-MAS (**S1 Fig**), as well as *in vivo* detectable macromolecule (and potential mobile lipid) contributions at 3.0, 2.8, 2.25, 2.05, 1.95, 1.67, 1.4, 1.3, 1.2, 0.9, and 0.85 ppm [28]. Only metabolite quantifications with Cramér-Rao Lower Bounds (CRLB) less than 50% were considered for further analysis. The non-suppressed water spectrum was used as reference for absolute quantifications, introducing a correction factor for water T2 in the fetal brain at birth based on literature clinical values at lower field (158 ms) [29,30]. We also compared our estimated total creatine levels with the values obtained from *ex vivo* HR-MAS of focus-microwave fixed samples (see **S1 Methods** section).

#### Spectral Pattern Classification

MRS data were further analyzed by pattern recognition analysis, according to a pipeline reported for mouse brain MR spectra [31]. This approach consists in developing classifiers predictive of group belonging (IUGR or Control) based on the MRS patterns. Spectra were individually processed with *jMRUI* v4.0 [25] (phase corrected and apodized, with 4Hz line broadening) and exported in *ASCII* format. The data were post-processed with *R* v3.0.1 [32] using scripts developed in-house, consisting in a linear baseline correction, referencing to the total choline peak (3.21ppm), and normalization of the region 4.25‒0.5 ppm to Unit Length (UL2), which was exported as a spectral vector. These spectral vectors (5 IUGR and 6 Control) were then fed into *SpectraClassifier* v3.1.2 [33], and concatenated either as Ctx+Hip vectors or Ctx+Str vectors, for classifier training. For each classifier, a fixed number of features (ppm positions corresponding to specific spectral vector point heights) were automatically extracted from the concatenated vectors. This step was carried out with the Sequential Forward Feature Selection (SFFS) method [34]. The features selected were used to generate classifiers by Fisher’s Linear Discriminant Analysis (LDA). For each classifier, a 2D latent space was defined by the projection of the canonical variables derived from the discriminant analysis. Altogether, three classifiers were generated for each set of concatenated vectors (Ctx+Hip and Ctx+Str), based on the number of features selected (1, 2 or 3).

### Statistical analysis

Statistical analysis of MRS quantitative data were performed with *IBM-SPSS* v19 (SPSS Inc, Chicago, IL, USA). MRS data from control and IUGR subjects were compared using a two-tailed unpaired Student's t-test (or a Mann-Whitney U test in case of abnormal sample distribution). A General Linear Model (GLM) was also used to adjust for confounding variables, such as brain temperature and the estimated relative contribution of each brain tissue studied to the respective MRS voxel; the litter and number of neighbor stillbirths were also used as confounding variables for a secondary GLM adjustment, to test for possible bias in our results related to the sub-cohort of animals selected for study. Results were considered significant at p-value <0.05. As for MRS data classification, the performance of each classifier was evaluated using bootstrapping with 1000 repetitions, which calculates the number of correctly classified cases. This module is integrated in *SpectraClassifier* v3.1.2 software.

## Results

### Perinatal effects of IUGR

#### Total animal cohort

At the time of partial uteroplacental vessel ligation (gestational day 25), the total number of fetuses per horn was uneven in all rabbit mothers. Thus, IUGR was induced in a total of 24 fetuses and 10 fetuses were used as control. At the time of birth (gestational day 30), 15 IUGR and 9 control fetuses were alive, indicating a higher rate of stillbirths in the IUGR group (n = 9, 37.5%) than in controls (n = 1, 10%), as expected in this model. The average weight of all IUGR and control newborns was 31.5±8.0g and 55.6±4.8g, respectively.

#### Animal cohort studied

**Fig 1:**
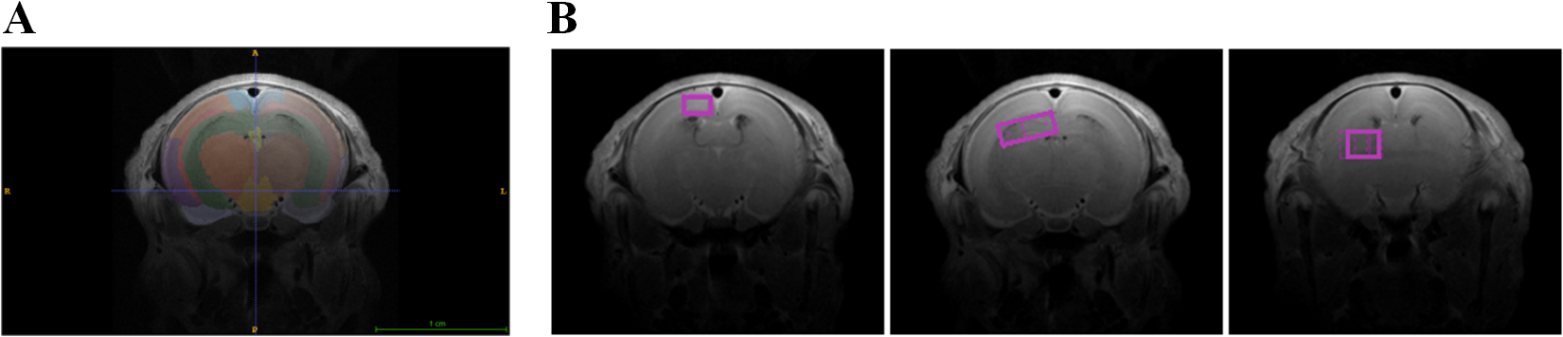
*In vivo* MRI with brain atlas registration and MRS voxel positions. **(A)** semi-automatic atlas registration on coronal T2-w image–legend: blue, cingulated cortex; orange and brown (outer), parietal cortex; purple, temporal cortex; grey, entorhinal cortex; red, subcortical white matter; green, hippocampus; orange and brown (inner), thalamus; yellow, corpus callosum; light orange, diencephalon. **(B)** MRS voxel positions on reference coronal T2-w MR images–from left to right: cortex, hippocampus, striatum.

In one of the litters, one IUGR newborn died before starting the MR study. Thus, a total of six controls and five IUGR newborns from three litters underwent MR examination within the first 8 h after delivery. The body weights of this subcohort (6 controls, 50–62g; 5 IUGRs, 29–36g) were representative of the main cohort (**Table 1)**, and the relative positions of those fetuses in the respective horn were not significantly different between IUGRs (0.5±0.2) and controls (0.7±0.3, p = 0.279). IUGR pups were significantly smaller than controls (-44% body weight) and had smaller placentas (-43% weight) (**Table 1**). The registration of *in vivo* MRI data with the digital atlas of the rabbit brain showed differences in the estimated average volumes of the total brain and specific brain tissues investigated (**Fig 1A**). These parameters were all significantly smaller compared to controls: total brain, -18%; cortex, -19%; hippocampus, -23%; and striatum, -21% (**Table 1**). The estimated contribution of each tissue studied to the respective MRS voxel (**Fig 1B**) was always >50%, although some significant differences were noticed in cortex and hippocampus due to overall smaller sizes in IUGR (**Table 1**). Specifically in the cortex region, the average relative composition of cortical tissue in the voxel was: cingulate cortex, 60%; frontal cortex, 6%; medial frontal cortex, 4%; and parietal cortex, 9%. Moreover, the animal body (rectal) temperatures remained constant throughout the MR studies, and were identical in both groups (around 35°C). However, the estimated brain temperatures in the three brain regions studied were consistently lower than the body temperature (around 31–32°C). Also, estimated local temperatures in IUGR brains were slightly lower than in controls, reaching significance only in the cortex region (**Table 1**).

**Table 1:** Animal cohort used for MR studies ‒ perinatal observations and MRI data. Controls vs IUGR parameters: average birth weight, placental weight, MRS voxel sizes per tissue, and estimated tissue contribution in voxel.

	Control (n = 6)	IUGR (n = 5)
Birth Weight (g)	56.0±4.9	31.4±3.4***
Placenta (g)	7.3±0.8	4.1±0.6***
Brain ^†^ (mm^3^)	1335.5±87.8	1100.6±28.7***
**Cortex:**		
Volume ^†^ (mm^3^)	476.2±29.9	387.2±24.1***
MRS ^†^ voxel (%)^¥^	79.4±7.7	78.5±7.5
Voxel temp. (°C)	31.5±1.1	29.7±1.1*
Body temp. (°C)	34.6±1.2	34.8±0.9
**Hippocampus:**		
Volume ^†^ (mm^3^)	173.3±15.2	134.2±6.0***
MRS ^†^ voxel (%)^¶^	70.1±7.8	62.2±4.9
Voxel temp. (°C)	31.9±1.1	31.0±1.6
Body temp. (°C)	34.4±1.3	34.7±0.6
**Striatum:**		
Volume ^†^ (mm^3^)	179.6±20.0	140.8±8.2**
MRS ^†^ voxel (%)^#^	61.4±5.2	50.8±7.0*
Voxel temp. (°C)	32.0±0.7	30.9±2.2
Body temp. (°C)	34.4±1.3	34.8±0.5

^†^ Estimated from semi-automated registration of T2-w MRI sections with digital rabbit brain atlas.

^¥^ Cingulate cortex: control, 63.8±7.9%; IUGR, 56.6±8.5%.

^¶^ Sub-cortical white matter: control, 19.5±4.2%; IUGR, 18.2±5.9%.

^#^ Basal ganglia (including striatum): control, 67.5±8.9%; IUGR, 65.1±7.0%. Student's t-Test

* p<0.05

** p<0.01

*** p<0.001.

### MRS quantification

**Fig 2:**
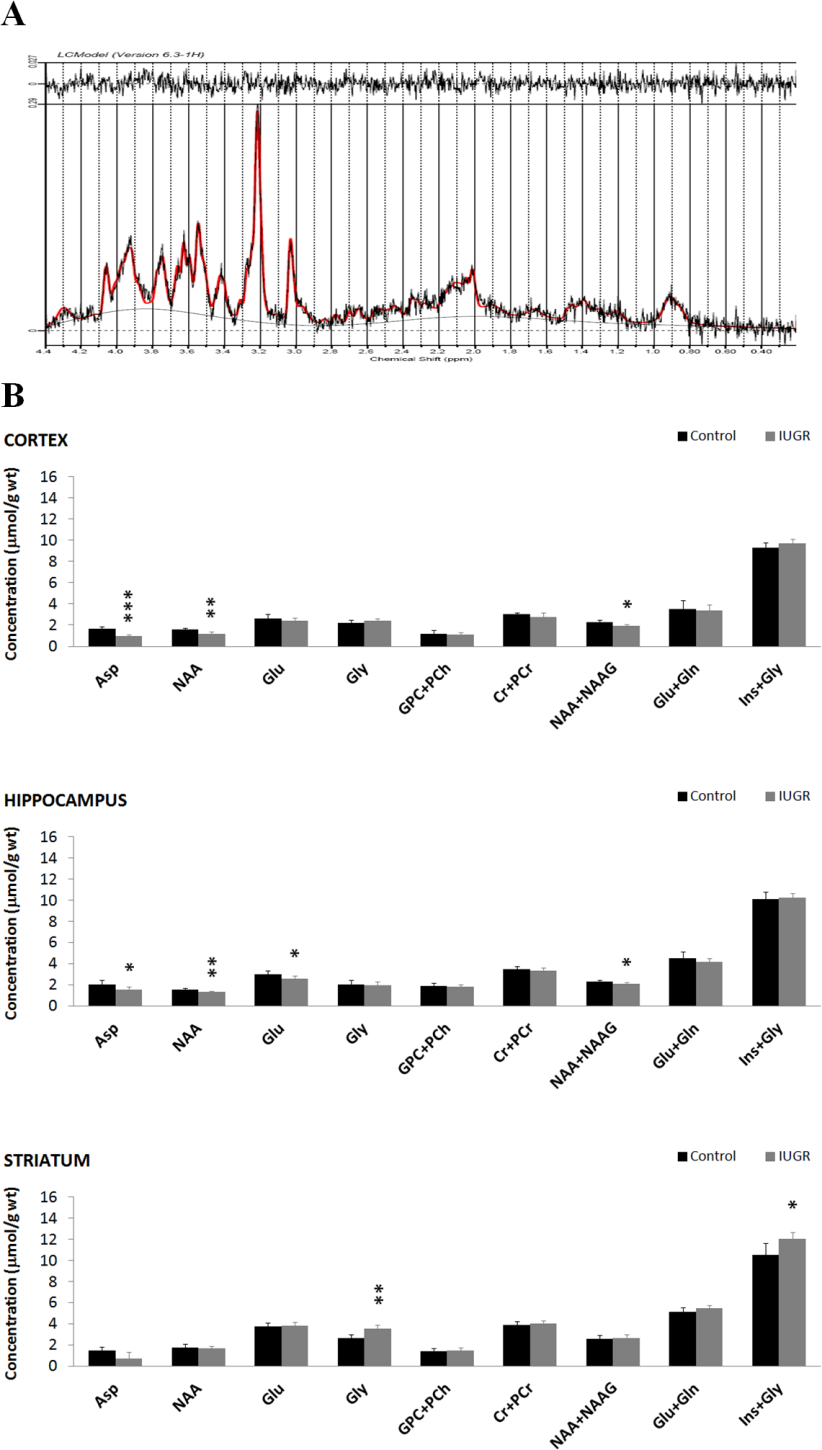
MRS quantifications. **(A)** Representative MRS profile of the hippocampus in a control animal (non-apodized spectrum, in black), with LCM baseline (grey line), fitting (red line), and residuals (top). **(B)** Selected metabolite levels in different brain regions: cortex (top), hippocampus (center), and striatum (bottom). Student´s t-Test: *p<0.05; **p<0.01; ***p<0.001. Aspartate (Asp); creatine (Cr); phosphocreatine (PCr); phosphorylcholine (PCh); glycero-phosphorylcholine (GPC); glutamate (Glu); glycine (Gly); myo-inositol (Ins); N-acetylaspartate (NAA); N-acetyl-aspartyl glutamate (NAAG).

The general good quality of the MRS data (**Fig 2A**) was evaluated by the full width at half maximum (FWHM) of the non-suppressed water peak (average, 10.1±2.7 Hz; maximum detected, 15.7 Hz), and also the signal-to-noise ratio (SNR) based on the total choline peak at 3.21 ppm (average, 47.7±12.6; minimum detected, 31.4). Slight local differences in SNR and FWHM were noticeable across different tissue areas but not between IUGR and controls, as summarized in **S1 Table**. Moreover, the average concentrations of total creatine estimated in cortex (3.0±0.1 μmol/g, n = 6) and striatum (3.9±0.3 μmol/g, n = 6) of control animals were in good agreement with the values obtained *ex vivo* by HR-MAS analysis (3.2±0.5 and 3.7±0.1 μmol/g, respectively).

### Brain metabolite changes associated with IUGR

The quantitative analysis of our MRS data indicated tissue-specific, brain metabolite changes in IUGR brains compared to controls (**Fig 2B**). Our model fitting estimated lower levels of N-acetylaspartate (NAA) (and the mixed pool of NAA and N-acetyl-aspartyl glutamate, NAA+NAAG) and aspartate in the cortex and hippocampus of IUGR pups. Glutamate also showed a slight decrease in the hippocampus, which was borderline significant (p = 0.051). All these metabolite changes were not significantly detected in the striatum. Instead, this brain region showed a significant increase of estimated glycine levels in IUGR brains, which was also apparent in the total myo-inositol and glycine pool (**Fig 2B**). These metabolic changes mostly remained significant after accounting for local brain temperature and estimated tissue contribution in each voxel (**Table 2**). Moreover, litter and number of neighbor stillbirths in each gestational sac did not represent a source of bias for our analysis (**S2 Table**).

**Table 2:** Relative changes in MRS-detectable metabolite levels.

Metabolite	Change in IUGR (%)		
	Ctx	Hip	Str
Aspartate	-41.6 ***	-24.4 *	-22.7
NAA	-24.2 **	-16.4 *	-5.8
NAA + NAAG	-14.2	-10.5	+4.6
Glutamate	-6.7	-13.7	+0.6
Glycine	+8.6	-2.7	+34.2 *
myo-Inositol + Glycine	+4.7	+1.5	+14.2 *

GLM (adjusting by brain temperature and estimated tissue contribution to voxel)

*p<0.05

**p<0.01

***p<0.001.

### MRS pattern changes in IUGR

**Fig 3:**
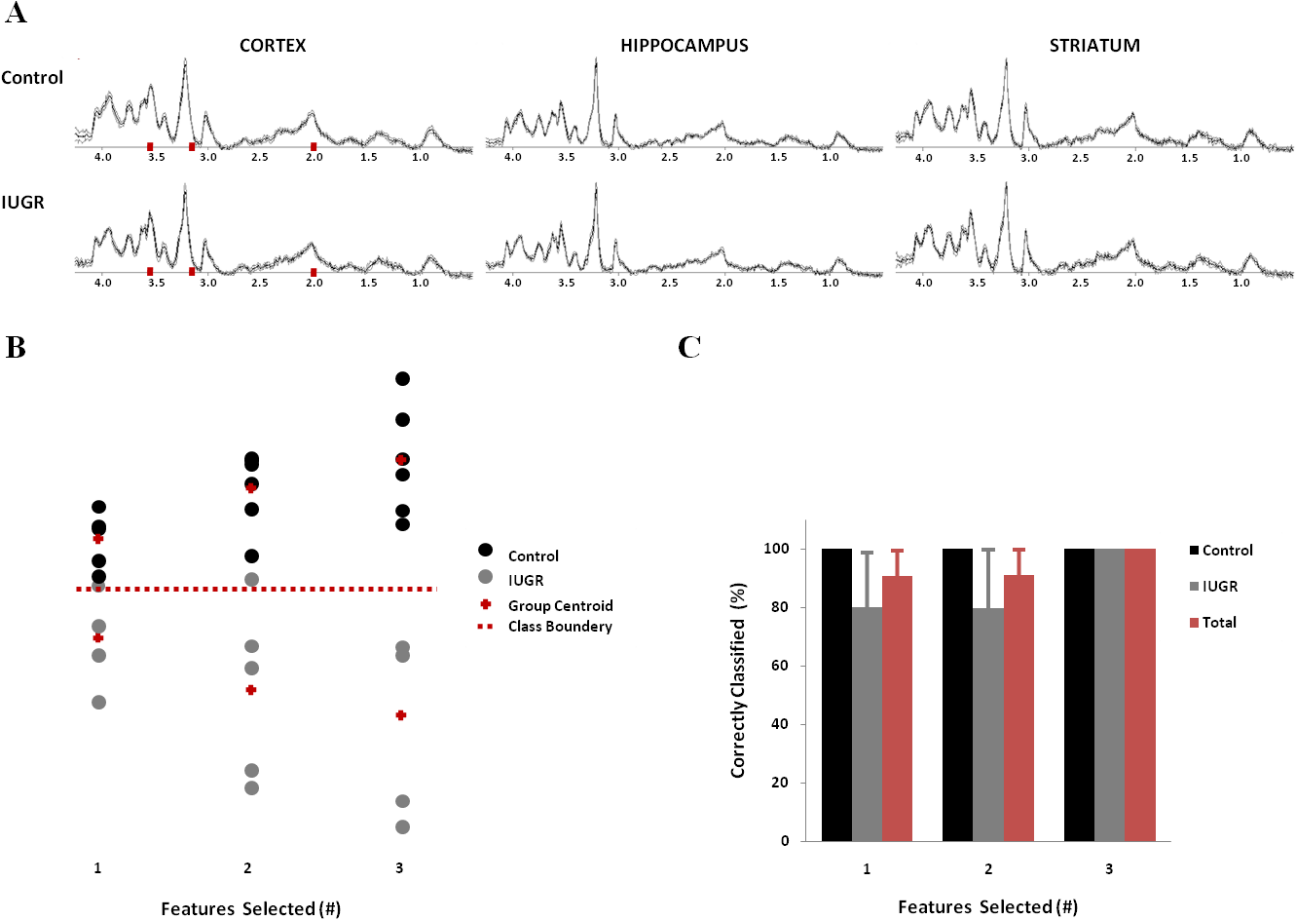
Classification of IUGR based on MRS pattern recognition analysis. **(A)** Average MRS vectors for Control (top) and IUGR (bottom), with overlaid standard deviation (grey lines), for each brain region—from left to right: cortex, hippocampus, striatum. Features objectively selected in cortex region highlighted in red (3.55, 3.15, 2.0 ppm: *Sequential Forward Feature Selection* method). **(B)** Latent space distribution of cortex spectral vectors based on LDA classifiers trained with different number of features (from left to right: 1, 2, and 3). **(C)** Evaluations of MRS classifiers. Three independent classifiers were generated for each combination of concatenated MRS vectors (cortex-hippocampus and cortex-striatum), according to the number of features. The features selected were the same for each vector concatenation and consistently from the cortex region only. Each classifier was evaluated by *Bootstrapping* (1000 repetitions).

When analyzing our MRS data as spectral vectors, using pattern recognition analysis (**Fig 3A**), we noticed that all features objectively selected for classification corresponded to the cortex region. Thus, the same results were consistently obtained when using either concatenated spectral vectors from cortex and hippocampus or from cortex and striatum. Classifiers developed only with 1 feature (3.55 ppm, **Fig 3B**) reached 90% accuracy in discriminating IUGR and control MRS patterns, as evaluated by bootstrapping (**Fig 3C**). In this case, the feature selected (3.55 ppm) corresponds to the mixed myo-inositol and glycine in the cortex. Although the estimated concentrations for both metabolites were not significantly different in this brain region between the two groups, a slight elevation in glycine was noticed in the IUGR group (+9%). Full predictive accuracy of the training set was reached by increasing the number of features selected up to 3 (**Fig 3B and 3C**), in which case the additional features objectively selected were 3.15 ppm (possible contributions from choline compounds or phenylalanine) and 2.0 ppm (NAA region ‒ decreased in IUGR, according to model fitting quantification), all corresponding to cortical spectral vectors.

## Conclusions

We have used a rabbit model of IUGR to assess *in vivo* brain structural and metabolic profile changes at the time of delivery. Low birth weight in these animals was associated with smaller brain sizes, slightly lower brain temperatures during anesthesia, and metabolite profile changes in different regions of the brain parenchyma. Specifically, we found apparent lower levels of aspartate and NAA in the cerebral cortex and hippocampus, and higher glycine in the striatum. MRS patterns analysis further suggests that the changes in cortex are the most prevalent.

The number of stillbirths and the birth weights in each group are in good agreement with the literature for this model [11,12,15], as well as the smaller brain sizes in IUGR pups [12,17]. A previous study on the same animal model of IUGR, showed predominant metabolite changes in the hemispheric regions at the time of birth, using frozen brain samples [17]. Specifically, the strongest differences between IUGR and control samples were found in asparagine (derived from aspartate), NAA, and pyroglutamic acid (a cyclic form of glutamate or glutamine), all decreased in IUGR brains [17]. These results are consistent with our data of lower apparent aspartate and NAA in both cortex and hippocampus, and slightly lower glutamate in the hippocampus.

A similar rabbit model of IUGR was also used to investigate brain metabolite changes in frozen tissue sections, collected from the same brain regions that we investigated [35]. However, some methodological differences between the two models may explain some of the different results obtained. In their study, occlusion was milder (30–40%) and performed earlier in gestation (day 21); and delivery was also later (day 32), which is in accordance with the higher birth weights reported by the authors (controls +16%; IUGRs +74%) [35]. This alternative approach showed significantly higher levels of glutamate (and dopamine) in all IUGR brain regions studied, along with lower GABA in the striatum; whereas we could not detect any significant changes in GABA but noticed a slight glutamate decrease in IUGR, mostly in the hippocampus. Moreover, previous studies comparing these two animal models of IUGR showed signs of cortical brain damage mostly in our model of IUGR (higher S100β expression), as well as very distinct patterns of cell proliferation in each brain region according to the timing and degree of the ligation [12]. This could explain to some extent the metabolic differences reported by the other group [35], supporting that the effect of IUGR on brain metabolism is dependent on the brain region but also on the degree and timing of the insult, according to the maturation stage of each brain region.

Our results also agree with recent clinical findings suggesting lower brain NAA levels in IUGR fetuses [9,10]. The stronger prevalence of metabolite pattern changes in the cortex region of IUGR brains is consistent with impaired neurodevelopmental performance detected in IUGR infants at 2 years of age, mostly related to frontal brain networking [36].

**Fig 4:**
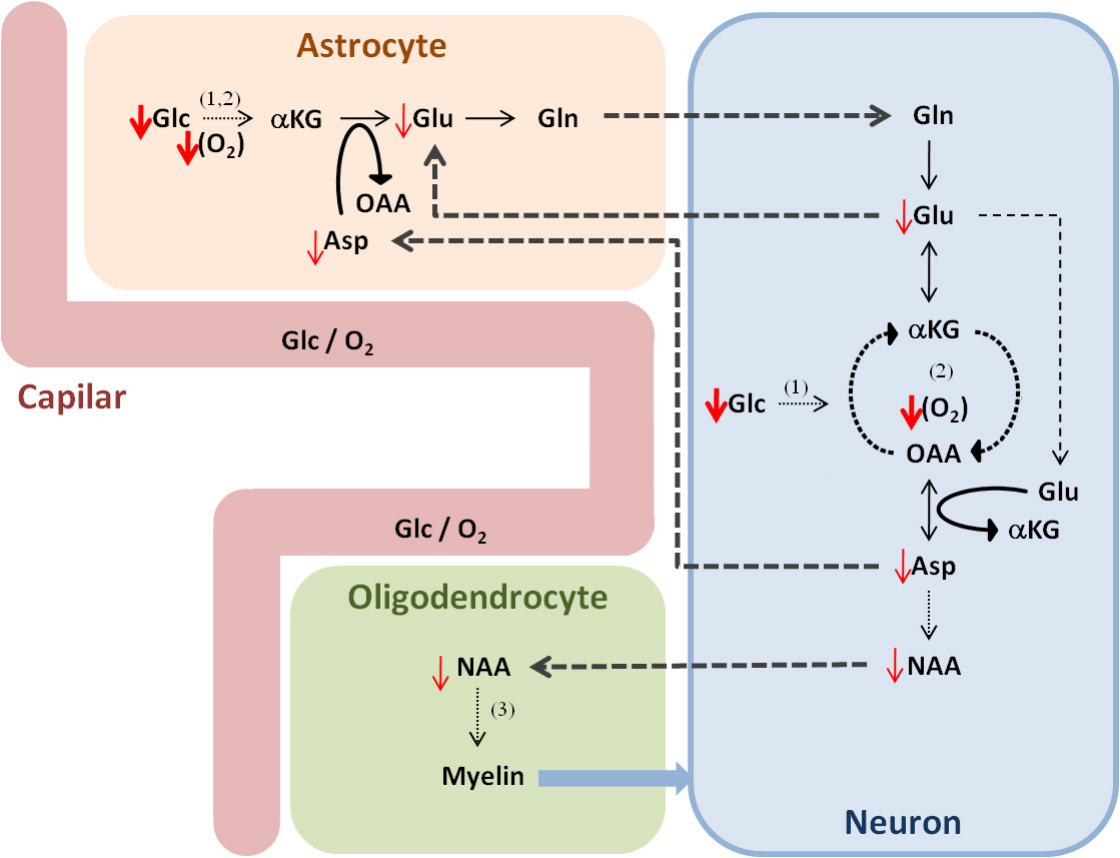
Metabolic model of IUGR impairment in cortex and hippocampus regions. Sustained hypoxemia (lower oxygen, O_2_) and under-nutrition (lower glucose, Glc) during IUGR lead to impaired brain metabolism, noticed *in vivo* as a decrease in products of aerobic metabolism (tricarboxylic acid cycle and oxidative phosphorylation (2)): Asp, NAA, and Glu. Basic metabolite shuttling between neurons and glial cells according to the literature [50,51,52]. (1) Glycolysis; (3) fatty acid synthesis; alpha-ketoglutarate (αKG); glucose (Glc); oxaloacetate (OAA).

As to the pathophysiologic explanation of our findings, the decrease of NAA (a neuronal marker and major precursor for myelin synthesis in oligodendrocytes) in the cortex and hippocampus of IUGR pups spatially correlates with the lower fractional anisotropy previously reported in this animal model at postnatal day 1 [14], which is indicative of decreased axonal packing in those regions—lower neuronal density, organization and/or myelinization. Then, the decrease of estimated aspartate levels and slight reduction of glutamate (two major excitatory neurotransmitters) would also agree with previous results with this model of impaired motor activity and olfactory function at postnatal day 1 [14], and anxiety, attention and memory problems at postnatal day 70 (pre-adolescent equivalent age) [15]: symptoms potentially related to impairments in parietal, frontal and cingulate cortex and hippocampus. Moreover, while the reduced brain/body temperatures during anesthesia agree with previous results in small animals [27,37,38], the lower cortical temperatures in the IUGR brainswould also agree with the clinical evidence of inefficient thermoregulation in low birth weight infants [39,40]. The latter observation suggests lower cortical metabolic activity, which could also explain the metabolite profile changes detected in this region. Altogether, these changes indicate impaired cellular metabolism due to sustained hypoxemia, under-nutrition, and apparent enhanced brain hypothermia in IUGR, most noticeable in products of mitochondrial aerobic metabolism in cortical and hippocampal regions (**Fig 4**). This is in agreement with previous results with this model [17] as well as with recent clinical findings [9,10].

The different profile of metabolite changes detected in the striatum (increased estimated levels of glycine and mixed pool of myo-inositol and glycine) compared to cortex and hippocampus, could be linked to different metabolic requirements in this region, where myelinization occurs earlier than in cortical regions. Since previous studies in rabbits showed acute injuries in the basal ganglia and thalamus due to prenatal hypoxia [41], the significant increase of estimated glycine levels (~10-fold higher in the normal fetal rabbit brain than in young animals [42]) could be a response to hypoxia during IUGR. This would agree with other studies showing a cytoprotective role for glycine [43,44], specifically by inhibiting apoptosis in brain cells during glucose and oxygen deprivation [45], which could in turn explain the apparent lower brain damage in the basal ganglia compared to cortex, in this model of IUGR [12]. Moreover, our results agree with recent clinical observations of an increased mixed pool of myo-inositol and glycine (relative to total choline) in the basal ganglia of fetuses with severe congenital heart disease, which is considered a model of brain development under chronic hypoxia [46].

Moreover, since the first feature objectively selected for MRS pattern classification was consistently 3.55 ppm, in the cortex region, this suggests that glycine, and/or myo-inositol, may also be altered in this region during IUGR but not readily quantifiable by model fitting. Accordingly, the estimated glycine levels were slightly elevated in IUGR cortex (+9%). Although selecting additional features for classification ultimately leads to classifier overtraining (features selected <1/3 spectral vectors available [47,48]), it is interesting to notice that those include 2.0 ppm and 3.15 ppm. While the former correlates with the NAA peak and therefore agrees with the quantitative differences estimated between groups, we found no significant metabolite differences around 3.15 ppm, i.e. estimated phenylalanine or choline compounds. Although we could detect free choline in our preliminary tissue extract NMR experiments, we did not select it for quantification (in agreement with the literature [28]) due to its much lower abundance *in vivo* compared with glycero-phosphocholine and phosphoryl choline, and also its overlap with phosphorylethanolamine.

This study has relevant clinical implications, since the brain metabolic changes detected support previous findings in fetal brain metabolite levels during IUGR [9,10] and correlate well with associated structural changes and neurodevelopment problems. The *in vivo* detection of these metabolites suggests their potential role as non-invasive biomarkers for the perinatal clinical diagnosis/monitoring of IUGR and other related diseases.

We acknowledge some limitations in our work. We cannot rule out possible mixed effects of anesthesia and (non-physiologic) brain hypothermia, and/or an acute response to the conditions outside the uterus in our model, which can impact brain metabolism. However, both IUGR and controls underwent the same procedures. Also, due to the small size and asymmetry of the different brain tissues, we were not able to fully position each MRS voxel within the respective tissue, although we assured their major relative contribution compared to nearby tissues. Then, selecting a shorter repetition time than in other small animals studies [28,49] was a compromise to acquire data in a timely manner, due to the difficulty in keeping newborn rabbits anesthetized for long periods. Although this induced some T1 saturation in our signals, the total creatine levels were similar to those obtained *ex vivo*; and most importantly, this should not affect the comparison between IUGR and controls. Moreover, the model basis sets for macromolecule fitting were taken from standard references and visual inspection of our data. Improving this performance would required metabolite suppression techniques [28], which were not available in our case. Still, no significant differences were detected in estimated macromolecule (or mobile lipid) contributions between IUGR and controls.

The stronger aspects of this work rely on the well characterized animal model of IUGR used that reproduces many clinical features of this disease [12,13], as well as the *in vivo* assessment of brain metabolite changes at the time of birth, which complements previous studies *post-mortem* with this model [17,35]. We have used MRS with high spatial resolution (voxel volume ~10 μl) and an echo time about 2-fold shorter than typically available in the clinic (≥20 ms). Thus, by carefully positioning the newborns in the animal holder and keeping them stably anesthetized (not trivial with newborn rabbits), we acquired good quality MRS data that enable a timely detection of low concentrated brain metabolites in different brain regions, reaching statistical significances with a relatively small animal cohort. Additionally, we used two independent approaches for MRS analysis, based on metabolite quantification and spectral pattern classification. This re-enforced our results since the two techniques are complementary, and the stronger metabolite differences estimated between groups do not necessarily represent the best spectral pattern features for classification.

To conclude, a well established animal model of IUGR showed differential metabolic profile changes in cortical and subcortical brain regions, with the most prevalent changes noticed in the cortex. This is consistent with postnatal neurobehavioral impairments reported in children and provides insight to the origin of the MRS changes recently reported in small fetuses at term. Future studies with this animal model of IUGR, or others, should investigate the longitudinal brain metabolic changes that parallel structural alterations, to help understanding the extent of IUGR from a biochemical point of view and provide additional biomarkers for its detection at different stages and/or monitor response to therapy.

## Supporting Information

S1 Fig *Post-mortem* NMR data from cortex sections of control animals. (Top) high-resolution NMR spectrum of metabolite extracts from frozen tissue samples, initially obtained after sacrificing the animal by decapitation. **(Bottom)** HR-MAS spectrum of chopped tissue from frozen tissue samples, initially obtained after sacrificing the animal by focused microwave irradiation. The metabolite assignments displayed are based on literature values, as detailed in **S1 Results**: 1, ascorbate; 2, lactate; 3, myo-inositol; 4, phosphorylethanolamine; 5 mixed pool total creatine/aspartate; 6, mixed pool glutamine/glutamate/glutathione; 7, glycine; 8, taurine; 9, glycero-phosphocholine; 10, phosphoryl choline; 11, phenylalanine; 12, total creatine (a, phosphocreatine; b, creatine); 13, glutathione; 14, gamma-aminobutyric acid (GABA); 15, aspartate; 16, mixed pool N-acetylaspartate/aspartate; 17, N-acetylaspartate (NAA); 18, glutamine; 19, glutamate; 20, N-acetyl-aspartyl glutamate (NAAG); 21, acetate; 22, alanine; 23, beta-hydroxybutyrate; 24, valine; 25, mixed pool glucose and scyllo-inositol (singlet 3.34 ppm); 26, mixed pool lactate/threonine. * methanol contamination from extraction.
(TIF)

S1 Methods Metabolite basis sets for *in vivo* MRS. (DOCX)

S1 Results Metabolite baseline of the fetal/newborn rabbit brain. (DOCX)

S1 Table MRS quality parameters in each tissue. MRS voxel: Ctx, cortex; Hip, hippocampus; Str, striatum. No significant differences were detected in the width at half maximum (FWHM) of the non-suppressed water peak, nor in the signal-to-noise ratio (SNR) based on the total choline peak. Student's t-Test: * p<0.05.
(DOCX)

S2 Table Effect of litter and stillbirths on metabolite levels. Brain MR metabolite changes between IUGR and control newborns are independent of the litter and proximity of stillbirths at birth. P values corresponding to GLM analysis of metabolite changes (Table 2), using litter and number of neighbor stillbirths as confounding variables. Significant changes highlighted in bold. Student's t-Test: *p<0.05; **p<0.01; ***p<0.001.
(DOCX)
