# Supplementary figures and images for: In Vivo Detection of Perinatal Brain Metabolite Changes in a Rabbit Model of Intrauterine Growth Restriction (IUGR)
Source: PLoS One. 2015 Jul 24;10(7):e0131310. doi: 10.1371/journal.pone.0131310 (PMC4514800; doi:10.1371/journal.pone.0131310)

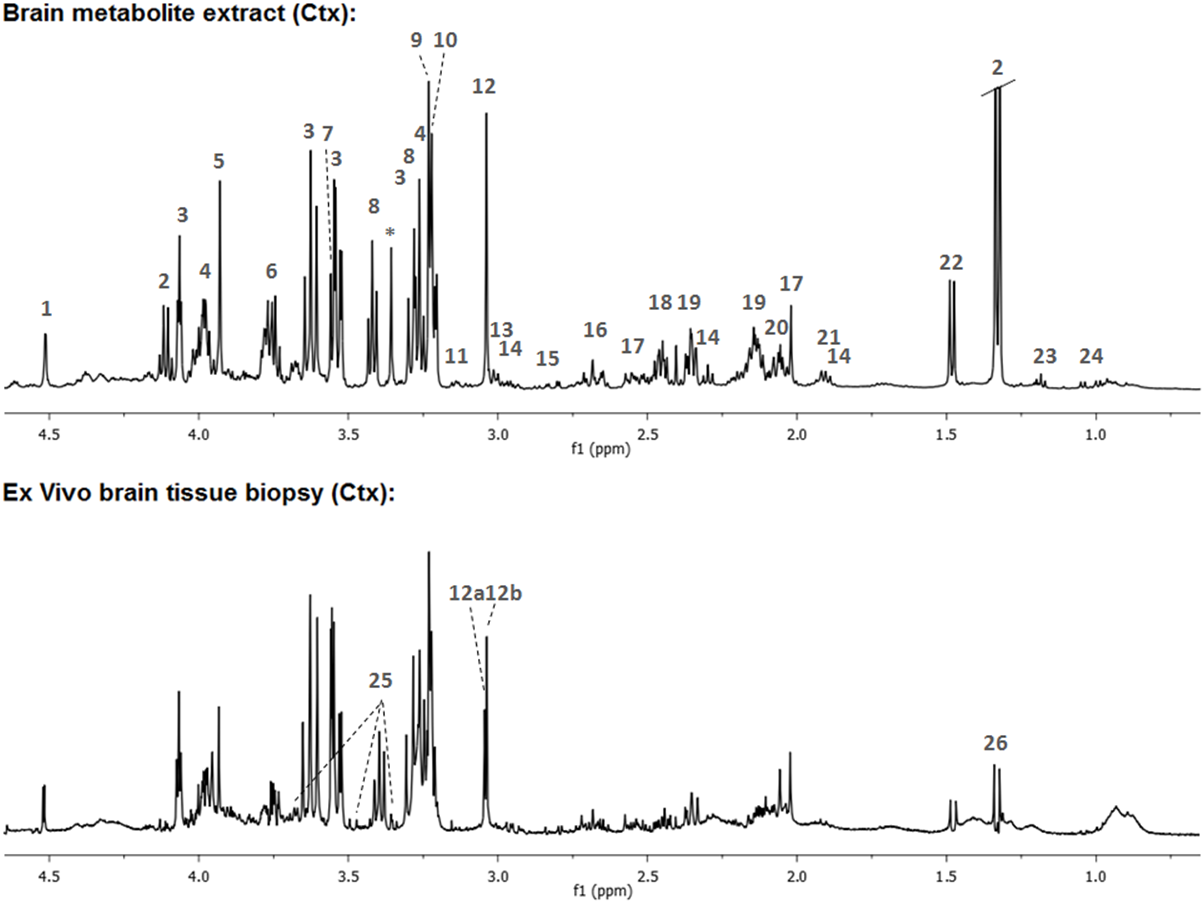

Supplement: S1 Fig — (Top) high-resolution NMR spectrum of metabolite extracts from frozen tissue samples, initially obtained after sacrificing the animal by decapitation. (Bottom) HR-MAS spectrum of chopped tissue from frozen tissue samples, initially obtained after sacrificing the animal by focused microwave irradiation. The metabolite assignments displayed are based on literature values, as detailed in S1 Results: 1, ascorbate; 2, lactate; 3, myo-inositol; 4, phosphorylethanolamine; 5 mixed pool total creatine/aspartate; 6, mixed pool glutamine/glutamate/glutathione; 7, glycine; 8, taurine; 9, glycero-phosphocholine; 10, phosphoryl choline; 11, phenylalanine; 12, total creatine (a, phosphocreatine; b, creatine); 13, glutathione; 14, gamma-aminobutyric acid (GABA); 15, aspartate; 16, mixed pool N-acetylaspartate/aspartate; 17, N-acetylaspartate (NAA); 18, glutamine; 19, glutamate; 20, N-acetyl-aspartyl glutamate (NAAG); 21, acetate; 22, alanine; 23, beta-hydroxybutyrate; 24, valine; 25, mixed pool glucose and scyllo-inositol (singlet 3.34 ppm); 26, mixed pool lactate/threonine. * methanol contamination from extraction. (TIF) [file pone.0131310.s001.tif]
