# Supplementary material for: In Vivo Detection of Perinatal Brain Metabolite Changes in a Rabbit Model of Intrauterine Growth Restriction (IUGR)
Source: PLoS One. 2015 Jul 24;10(7):e0131310. doi: 10.1371/journal.pone.0131310 (PMC4514800; doi:10.1371/journal.pone.0131310)
Supplement: S1 Methods — (DOCX) [file pone.0131310.s002.docx]

**S1 METHODS**

**Metabolite basis sets for *in vivo* MRS**

Control animals were obtained from two additional mothers (not reported in the main study), which were submitted to the same chirurgical procedure described in the methods section "Animal model of IUGR". After delivery, three control animals (>50g) were obtained from each mother (groups A and B). Animals in group A were sacrificed by decapitation after delivery, their brains were removed, and hemisphere and basal ganglia regions were separated, frozen in liquid nitrogen and stored at -80⁰C until further analysis by high-resolution NMR (A). Animals in group B were instantly sacrificed (1.4s) during the first 8h of life by brain focused microwave irradiation (Microwave Fixation System TMW-6402-C 5KHz, Muromachi Kikai Co. Ltd., Tokyo, Japan), as reported before in rats and mice [[1](#_ENREF_1), [2](#_ENREF_2)]. The brains were then removed and samples were collected from the prefrontal cortex and basal ganglia, and stored at -80⁰C until further study by *ex vivo* HR-MAS (B).

**A) high-resolution NMR**

Brain tissue samples (90-100 mg each) from decapitated animals were individually submitted to a dual phase extraction [[3](#_ENREF_3)]. Tissue samples at -80 °C were ground on a mortar in the presence of liquid N_2_ to avoid thawing. The resulting powder was kept at -20 °C till extraction. Samples were sequentially vortexed in previously iced-cold solvents methanol/water (5:1), chloroform, and chloroform/water (1:1), and then phase separated by centrifugation (1000g, 30’, 4 °C). The polar part (methanol/water) was lyophilized and stored at -80 °C. The apolar fractions (CHCl_3_) were dried by speed-vac and subsequently stored at the same temperature in 100% CDCl_3_ with 0.03% TMS, to avoid oxidation. For the NMR measurements, the polar samples were dissolved in a phosphate buffer with TSP (0.5 mM), NaN_3_ (0.01%) and D_2_O (100%), and pH adjusted to 7.4 with HCl [[4](#_ENREF_4)]. All spectra were recorded at 300 K with a Bruker Avance III 500 MHz spectrometer equipped with a 5 mm broadband room temperature TBI probe (Bruker Biospin, Rheinstetten, Germany). ^1^H noesy1D spectra were collected using 256 scans, 64K data points, 24 ppm of spectral width, 4 s relaxation delay and 10 ms of mixing time [[5](#_ENREF_5)]. Water presaturation was applied during both relaxation and mixing times. To aid in metabolite identification 2D *J*-resolved (64 transients and 80 increments), ^1^H,^1^H-TOCSY and ^13^C-HSQC experiments were acquired for selected samples, using 256-512 increments, 64-128 transients and 1.5 s for relaxation delay.

**B) *ex vivo* HR-MAS**

Tissue samples from focused microwave fixated brains were used for HR-MAS analysis, as reported previously [[2](#_ENREF_2)]. Briefly, each frozen sample (15-25 mg) was sectioned with a scalpel and inserted on a HR-MAS micro-rotor (HZ05538 BL4 12µL Teflon spacer, *Cortecnet*, Paris, France). Deuterium dioxide (100%, *Carlo Erba Réactifs-SDS*, Val de Reuil, France) prepared with 0.9% NaCl was added to each rotor and homogeneized wih a 1ml syringe. Finally, the rotors were capped and studied by HR-MAS on a 400 MHz spectrometer (*Bruker BioSpin*, Wissembourg, France) equipped with a multi-nuclear HR-MAS resonance probe. The studies were carried out at room temperature and using the standard spinning rate for our field: 3000 Hz. ^1^H spectra were acquired for all samples with 10 ppm spectral width and 16K data points, using two sequences: a standard *pulse-acquire* (*zg*), with 10 s relaxation delay and 8 scans; and a *CPMG* sequence with water presaturation [[6](#_ENREF_6)], with 4 s relaxation delay and 128 scans.

**C) Data processing**

High resolution NMR and ex vivo HR-MAS data were processed with *Mnova* v8.1 (Mestrelab Research, Santiago de Compostela, Spain). A line broadening of 1.0 Hz was applied to each FID, which was zero-filled to double the number of Fourier domain points. Spectra were phased manually and the baseline corrected automatically. Chemical shifts were referenced internally to the CH_3_ resonance of alanine at 1.47 ppm, and spectral peak assignments were based on literature values [[7](#_ENREF_7)]. The metabolites more readily identifiable by both techniques (**S1** **Fig.**) were included in the simulated basis sets for quantification of *in vivo* brain MRS data. Additionally, the concentration of total creatine (3.03 ppm) was estimated from the *zg* acquired HR-MAS data. Thus, the water and creatine peaks were deconvoluted and their areas (A) used as follows:

$\left[Metab\right]=\frac{A_{Metab}}{\eta_{Metab}}\times\frac{\eta_{Water}}{A_{Water}}\times\frac{10^{6}}{M_{Water}}$ (1)

where [Metab]= metabolite concentrations in μmol/g water; n= number of magnetically equivalent protons in the resonance used (water, 2; creatine, 3); M=molar mass (water, 18 g/mol). This method is a slight modification of the approaches described in references [[2](#_ENREF_2), [8](#_ENREF_8)]; T2 and T1 correction factors were disregarded since no echo-time was used and the TR selected was long enough to prevent apparent saturation effects in the signals (visual inspection).

**REFERENCES**

1. de Graaf, R.A., et al., *In situ 3D magnetic resonance metabolic imaging of microwave-irradiated rodent brain: a new tool for metabolomics research.* J Neurochem, 2009. **109**(2): p. 494-501.

2. Simões, R.V., et al., *1H-MRSI pattern perturbation in a mouse glioma: the effects of acute hyperglycemia and moderate hypothermia.* NMR Biomed, 2010. **23**(1): p. 23-33.

3. Le Belle, J.E., et al., *A comparison of cell and tissue extraction techniques using high-resolution 1H-NMR spectroscopy.* NMR Biomed, 2002. **15**(1): p. 37-44.

4. Beckonert, O., et al., *Metabolic profiling, metabolomic and metabonomic procedures for NMR spectroscopy of urine, plasma, serum and tissue extracts.* Nat Protoc, 2007. **2**(11): p. 2692-703.

5. Nicholson, J.K., et al., *750 MHz 1H and 1H-13C NMR spectroscopy of human blood plasma.* Anal Chem, 1995. **67**(5): p. 793-811.

6. Meiboom, S. and D. Gill, *Modified spin-echo method for measuring nuclear relaxation times.* Rev. Sci. Instrum., 1958. **29**(8): p. 688-691.

7. Govindaraju, V., K. Young, and A.A. Maudsley, *Proton NMR chemical shifts and coupling constants for brain metabolites.* NMR Biomed, 2000. **13**(3): p. 129-53.

8. Bolan, P.J., et al., *In vivo quantification of choline compounds in the breast with 1H MR spectroscopy.* Magn Reson Med, 2003. **50**(6): p. 1134-43.
