# Supplementary material for: In Vivo Detection of Perinatal Brain Metabolite Changes in a Rabbit Model of Intrauterine Growth Restriction (IUGR)
Source: PLoS One. 2015 Jul 24;10(7):e0131310. doi: 10.1371/journal.pone.0131310 (PMC4514800; doi:10.1371/journal.pone.0131310)
Supplement: S1 Results — (DOCX) [file pone.0131310.s003.docx]

**S1 RESULTS**

**Metabolite baseline of the fetal/newborn rabbit brain**

We identified up to 22 metabolites in control rabbit brain samples, using either high resolution NMR (tissue extracts) or HR-MAS (fixed brains), including: ascorbate, alanine, aspartate (Asp), creatine (Cr), phosphocreatine (PCr), phosphorylcholine (PCh), glycero-phosphorylcholine (GPC), gamma-aminobutyric acid (GABA), glucose, phenylalanine, glutamate (Glu), glutamine, glycine (Gly), glutathione, lactate, myo-inositol (Ins), N-acetylaspartate (NAA), N-acetyl-aspartyl glutamate (NAAG), Scyllo-Inositol, taurine, threonine, and phosphorylethanolamine (**S1** **Fig. -** metabolite assignments based on literature values [[1](#_ENREF_1)]). High levels of lactate and alanine were detectable in all tissue extracts, due to *post-mortem* anaerobic metabolism from the time animal sacrifice until snap-freezing the samples. In fixed tissue samples this was not observed since the focused microwave irradiation immediately halts cellular metabolism [[2](#_ENREF_2)]. Thus, both phosphocreatine and creatine could be detected in this case, whereas only a creatine pool was observed in tissue extracts. The estimated levels of total creatine in control brains were 3.2±0.5 µmol/g in cortex, and 3.7±0.1 µmol/g in striatum.

**REFERENCES**

1. Govindaraju, V., K. Young, and A.A. Maudsley, *Proton NMR chemical shifts and coupling constants for brain metabolites.* NMR Biomed, 2000. **13**(3): p. 129-53.

2. de Graaf, R.A., et al., *In situ 3D magnetic resonance metabolic imaging of microwave-irradiated rodent brain: a new tool for metabolomics research.* J Neurochem, 2009. **109**(2): p. 494-501.
