# Supplementary material for: In Vivo Detection of Perinatal Brain Metabolite Changes in a Rabbit Model of Intrauterine Growth Restriction (IUGR)
Source: PLoS One. 2015 Jul 24;10(7):e0131310. doi: 10.1371/journal.pone.0131310 (PMC4514800; doi:10.1371/journal.pone.0131310)
Supplement: S1 Table — MRS voxel: Ctx, cortex; Hip, hippocampus; Str, striatum. No significant differences were detected in the width at half maximum (FWHM) of the non-suppressed water peak, nor in the signal-to-noise ratio (SNR) based on the total choline peak. Student's t-Test: * p<0.05. (DOCX) [file pone.0131310.s004.docx]

**S1 Table.** **MRS quality parameters in each tissue.** MRS voxel: Ctx, cortex; Hip, hippocampus; Str, striatum. No significant differences were detected in the width at half maximum (FWHM) of the non-suppressed water peak, nor in the signal-to-noise ratio (SNR) based on the total choline peak.

|  | **Ctx** | | **Hip** | | **Str** | |
| --- | --- | --- | --- | --- | --- | --- |
|  | **Control** | **IUGR** | **Control** | **IUGR** | **Control** | **IUGR** |
| FWHM (Hz) | 12.7±1.2 | 12.3±2.4 | 8.0±2.2 | 8.8±2.9 | 8.2±1.3 | 9.8±1.0 |
| SNR (Cho) | 46.0±6.2 | 43.3±11.3 | 61.7±14.2 | 55.5±14.5 | 41.3±3.5 | 37.2±4.2 |

Student's t-Test: * p<0.05.
