# Supplementary material for: In Vivo Detection of Perinatal Brain Metabolite Changes in a Rabbit Model of Intrauterine Growth Restriction (IUGR)
Source: PLoS One. 2015 Jul 24;10(7):e0131310. doi: 10.1371/journal.pone.0131310 (PMC4514800; doi:10.1371/journal.pone.0131310)
Supplement: S2 Table — Brain MR metabolite changes between IUGR and control newborns are independent of the litter and proximity of stillbirths at birth. P values corresponding to GLM analysis of metabolite changes (Table 2), using litter and number of neighbor stillbirths as confounding variables. Significant changes highlighted in bold. Student's t-Test: *p<0.05; **p<0.01; ***p<0.001. (DOCX) [file pone.0131310.s005.docx]

**S2 Table.** **Effect of litter and stillbirths on metabolite levels.** Brain MR metabolite changes between IUGR and control newborns are independent of the litter and proximity of stillbirths at birth. P values corresponding to GLM analysis of metabolite changes (Table 2), using litter and number of neighbor stillbirths as confounding variables. Significant changes highlighted in bold.

| **Metabolite** | **Control vs IUGR (GLM, p values)** | | |
| --- | --- | --- | --- |
|  | **Ctx** | **Hip** | **Str** |
| Aspartate | .000*** | .029* | .111 |
| NAA | .002** | .003** | .546 |
| NAA + NAAG | .021* | .017* | .568 |
| Glutamate | .453 | .051 | .911 |
| Glycine | .231 | .814 | .004** |
| myo-Inositol + Glycine | .125 | .689 | .025* |

Student's t-Test: *p<0.05; **p<0.01; ***p<0.001.
